# Role of Humoral versus Cellular Responses Induced by a Protective Dengue Vaccine Candidate

**DOI:** 10.1371/journal.ppat.1003723

**Published:** 2013-10-31

**Authors:** Raphaël M. Zellweger, Robyn Miller, William E. Eddy, Laura J. White, Robert E. Johnston, Sujan Shresta

**Affiliations:** 1 La Jolla Institute for Allergy & Immunology, La Jolla, California, United States of America; 2 Global Vaccines Inc., Research Triangle Park, North Carolina, United States of America; Erasmus Medical Center, Netherlands

## Abstract

With 2.5 billion people at risk, dengue is a major emerging disease threat and an escalating public health problem worldwide. Dengue virus causes disease ranging from a self-limiting febrile illness (dengue fever) to the potentially fatal dengue hemorrhagic fever/dengue shock syndrome. Severe dengue disease is associated with sub-protective levels of antibody, which exacerbate disease upon re-infection. A dengue vaccine should generate protective immunity without increasing severity of disease. To date, the determinants of vaccine-mediated protection against dengue remain unclear, and additional correlates of protection are urgently needed. Here, mice were immunized with viral replicon particles expressing the dengue envelope protein ectodomain to assess the relative contribution of humoral versus cellular immunity to protection. Vaccination with viral replicon particles provided robust protection against dengue challenge. Vaccine-induced humoral responses had the potential to either protect from or exacerbate dengue disease upon challenge, whereas cellular immune responses were beneficial. This study explores the immunological basis of protection induced by a dengue vaccine and suggests that a safe and efficient vaccine against dengue should trigger both arms of the immune system.

## Author Summary

Dengue virus is an escalating public health threat for over 2.5 billion people worldwide. The disease caused by dengue virus ranges from mild (dengue fever) to lethal (dengue hemorrhagic fever, dengue shock syndrome). To date, there is no cure or vaccine for dengue. One of the challenges to developing a safe and efficient dengue vaccine is that antibodies, usually induced by vaccines to protect the host from re-infection, can increase the severity of dengue disease if they are not present in sufficient amounts to neutralize the virus. An efficient vaccine is urgently needed to slow down the progression of dengue disease, but little is known about the way the immune system protects the body against dengue re-infection. Using a protective vaccine candidate for dengue, the present study evaluates in mice the relative contribution of T cells and antibodies to protection against dengue. We show that the antibody component of an immune response that is overall protective had the ability, when isolated from the other components of the immune system, to either decrease or increase viral burden, whereas T cells reduced viral burden in all situations tested. Our results suggest that vaccine development efforts should focus on approaches that induce both T cell and antibody responses against dengue virus.

## Introduction

The four serotypes of dengue virus (DENV1-4) are mosquito-borne and cause a spectrum of diseases ranging from a self-limiting flu-like illness (dengue fever, DF) to the potentially lethal dengue hemorrhagic fever/dengue shock syndrome (DHF/DSS) [1]. DENV is endemic in more than 100 countries [2] and 2.5 billion people worldwide are at risk of infection, mostly in tropical and subtropical regions [3]. It is estimated that 390 million cases of DENV infection occur annually, of which 96 million are apparent, 500,000 are severe and 20,000 are fatal [4].

The more severe disease resulting from DENV infection, DHF/DSS, usually occurs in individuals who have pre-existing dengue-reactive antibodies (Abs), acquired either from a previous infection with a heterologous DENV serotype or by passive transfer from an immune mother in the case of infants [5]. Based on these epidemiological observations, Halstead and colleagues hypothesized that sub-protective levels of DENV-specific Abs may amplify viral infection and thus exacerbate disease, a phenomenon termed antibody-dependent enhancement of infection (ADE) [6], [7]. We and another group have recently confirmed this hypothesis by demonstrating in mice that a sub-protective amount of anti-DENV Abs can turn a mild illness into a lethal disease upon infection with DENV [8], [9].

The potential risk of ADE represents a major challenge associated with the development of a safe vaccine against DENV [2]. A vaccine that induces sub-protective levels of anti-DENV Abs may not only be inefficient, but also potentially cause ADE-mediated severe dengue disease upon infection. In addition, despite the initial induction of a protective Ab response, the Ab levels could wane and reach ADE-causing concentrations some time after vaccination, as even protective anti-DENV Ab has the ability to cause ADE at lower concentrations [9]–[11].

Detecting neutralizing Ab in vitro may not accurately correlate with protection in vivo, as recently exemplified by the results of the phase IIb clinical trial of the most advanced dengue-vaccine candidate [12]. The vaccine candidate had only limited efficacy despite induction of a balanced neutralizing Ab response to all four serotypes. This infers the involvement of other branches of the immune system in protection against DENV. The role of T cells during re-infection is controversial, and often seen as minor [2] or pathogenic [13]. Accordingly, it is commonly accepted that the primary goal of dengue vaccination should be induction of neutralizing Ab responses, and that vaccine-induced T cell responses likely play only a secondary role in protection. However, there is a substantial lack of knowledge of the immune mechanisms involved in protection during successive DENV infections [14], [15]. Therefore, a better understanding of the relative role of the humoral versus cellular components of a vaccine-induced immune response to protection against dengue virus infection is urgently needed. The goal of our study was to assess the relative contribution of the humoral and cellular arms of the immune system in protection mediated by a dengue-vaccine candidate.

Venezuelan equine encephalitis virus (VEE) is an alphavirus that can be used as a vaccine expression vector in which the genes coding for the structural proteins are replaced by one or more transgenes [16]. The resulting viral replicon particles (VRP) induce high level expression of the transgenes in a single round of infection, but due to the absence of endogenous structural proteins, do not propagate further in the host [16]. VRP expressing HIV [17], influenza [18], or human cytomegalovirus [19] immunogens have been used safely in phase I vaccine trials in humans. VRP coding for DENV membrane (prM/M) and envelope (E) proteins have been previously used to immunize mice [20]. VRP immunization induced anti-DENV neutralizing Abs and protected suckling mice from lethal intracranial DENV challenge [20]. Recently, White and colleagues demonstrated that immunization with VRPs expressing the DENV-E protein ectodomain (E85-VRP) derived from each of the four DENV serotypes induced balanced neutralizing Ab responses to all four serotypes in mice and in macaques, and protected macaques from infection with DENV (White, unpublished observations) [21].

In the present study, VRP expressing the DENV2-E protein ectodomain (DENV2 E85-VRP) were used to immunize AG129 mice (type I and II IFN receptor-deficient mice in the 129/Sv genetic background), followed by challenge with DENV serotype 2 (DENV2) to assess the relative contribution of the cellular versus humoral components of a protective vaccine-induced immune response against DENV. AG129 were used because DENV replicates to high levels in these mice, and they represent the best-characterized animal model of DENV infection in which DHF/DSS-like disease can be induced. The results obtained using AG129 mice were confirmed in an adoptive transfer system. Wildtype (WT) mice were immunized with the DENV2 E85-VRP, and, subsequently, DENV2 E85-VRP primed WT T cells, B cells or serum were transferred into AG129 recipient mice prior to challenge. This transfer system allowed us to assess the contribution of the cellular and humoral components the DENV2 E85-VRP-induced immune response generated in WT mice. Due to their high sensitivity to DENV infection, the AG129 recipient mice served as a stringent challenge model to assess the contribution of the transferred cells or serum from WT mice.

Two rounds of immunization with DENV2 E85-VRP efficiently protected AG129 mice from challenge with DENV even when disease-enhancing amounts of Abs were administered at the time of challenge (ADE conditions). Mice were protected as early as five days after the second immunization, and remained protected for at least four weeks after immunization. Short-term protection was mainly mediated by CD8^+^ T cells, whereas long-term protection relied on CD8^+^ T cells to different degrees depending on the immunization schedule. These results were confirmed in a series of transfer experiments where T cells, B cells or serum from DENV2 E85-VRP vaccinated WT mice were transferred into AG129 mice before challenge. Transfer of DENV2 E85-VRP-primed WT T cells into naïve AG129 mice reduced viral load upon challenge with DENV. In contrast, transfer of DENV2 E85-VRP-immune serum had the potential to increase viral load. Transfer of DENV2 E85-VRP-primed WT B cells into naïve mice either reduced or increased viral load depending on the number of B cells transferred. These results demonstrate that, taken in isolation and at certain concentrations, the humoral component of a protective vaccine-induced immune response to DENV has the potential to exacerbate dengue disease, whereas in all conditions tested, the cellular immune response reduced viral load. This implies that a safe and protective vaccine against DENV should trigger both the cellular and humoral arms of the immune system rather than relying exclusively on induction of DENV-specific Abs.

## Results

### DENV2 E85-VRP immunization protects from DENV challenge and induces DENV-specific antibody

To assess whether immunization with DENV2 E85-VRP would protect AG129 mice (lacking type I and II IFN receptors) against DENV, AG129 mice were immunized twice with DENV2 E85-VRP prior to challenge with DENV. AG129 were used because they are highly sensitive to DENV infection and can develop DHF/DSS-like lethal disease upon infection, and are the best characterized animal model of DENV infection to date. Using these mice, we have previously demonstrated ADE in vivo and confirmed that even a protective anti-DENV Ab can induce ADE at sub-protective concentrations [9]. The viral strain used for challenge in our study, S221, is a triple-plaque-purified clone isolated from a mouse-passaged DENV2 strain and has been previously described [22], [23]. This strain was used to challenge the mice in all our experiments, and for clarity will be referred to as “DENV” throughout the text. We have previously demonstrated that at the S221 challenge dose of 5×10^8^ genomic equivalents (GE), AG129 mice do not develop severe disease, but instead manifest neurological symptoms between day 11–14 after infection [9], [22]. In contrast, in the presence of sub-neutralizing amounts of anti-DENV Ab 2H2, antibody-mediated disease enhancement occurs and the same dose of virus (5×10^8^ GE) causes elevated viral RNA titers in the liver on day 3 and lethal DHF/DSS-like disease by day 4–5 [9]. Therefore, 5×10^8^ GE was chosen as the challenge dose for the present study, and we hypothesized that protective vaccination should reduce liver RNA viral titers and prevent neurological symptoms as well as death, whereas potential enhancement would be reflected by elevated liver titers and acute lethal disease around day 4–5.

**Figure 1:**
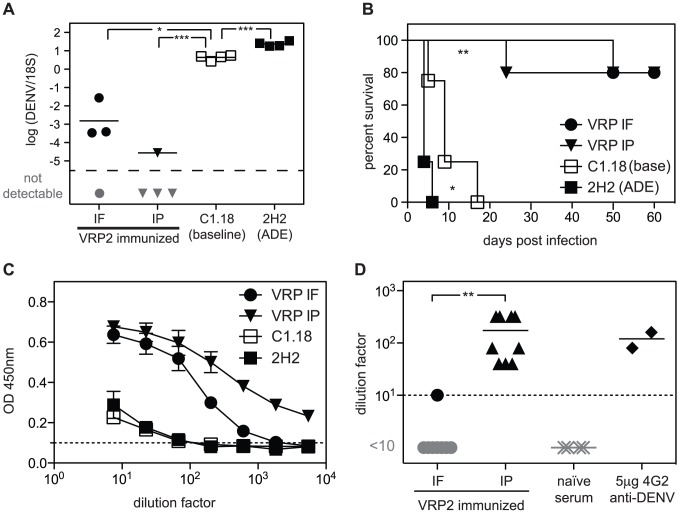
DENV2 E85-VRP-immunization protects from DENV challenge. AG129 mice were immunized with 1×10^6^ IU DENV2 E85-VRP either i.f. (black circles) or i.p. (black triangles) 14 and 5 days prior to challenge with 5×10^8^ GE DENV. Two groups of mice were not immunized prior to infection with DENV, one of which was infected in the presence of anti-DENV antibody 2H2, resulting in antibody-mediated enhancement of disease (black squares, ADE group) and the other one was infected in the presence of antibody C1.18 which is an isotype control of irrelevant specificity (white squares, baseline group). Viral RNA levels were measured in the liver 3 days after challenge (A) and survival was monitored (B). One day prior to challenge, serum DENV-specific IgG were measured by ELISA on DENV-coated plates (C) and the neutralization capacity of the serum was determined by PRNT_50_ (D). Each symbol depicts one mouse except in B where n = 4–5, and in C where each symbol represents the mean of 4 animals, P-values from two-tailed unpaired t-test with Welch's correction, confidence interval 95% (A, C, D) or Gehan-Breslow-Wilcoxon test (B), *P≤0.05, **P≤0.01, ***P≤0.001. The dotted line represents the limit of detection of the assay. Samples with undetectable levels of DENV2 RNA are represented in gray under the detection limit. As they have no numerical value, they were not taken into account to calculate the mean.

AG129 mice were immunized with 1×10^6^ infectious units (IU) of DENV2 E85-VRP either intraperitoneally (i.p.) or intra footpad (i.f.) 14 and 5 days prior to challenge with 5×10^8^ GE DENV on day 0. As non-vaccinated controls, two groups that were not immunized were challenged with 5×10^8^ GE DENV: one group in the presence of 15 µg of exogenous monoclonal anti-DENV Ab 2H2 given i.p. to cause Ab-mediated enhancement of infection (“ADE group”) and another group in the presence of 15 µg of C1.18, an isotype control Ab of irrelevant specificity (“baseline group”). Viral RNA in the liver was quantified by qRT-PCR on day 3 (figure 1A) and survival was monitored (figure 1B). The liver was chosen because high viral RNA levels in the liver on day 3 correlate with increased severity of disease and decreased survival [9]. Immunization through either the i.p. or i.f. route dramatically reduced viral RNA levels in the liver (figure 1A) and prevented death in 80% of immunized animals (figure 1B). As expected from our previous work [9], the ADE group had approximately 10-fold more viral RNA in the liver on day 3 and its survival was decreased relative to the baseline group. These results demonstrate that DENV2 E85-VRP immunization provides protection against DENV infection and disease, as measured by liver DENV titer and survival, respectively.

To start dissecting the immunological basis of this DENV2 E85-VRP-induced protection, the induction of Abs following immunization with DENV2 E85-VRP on day -14 and -5 was assessed in the serum of AG129 mice 1 day before challenge with DENV. Virus-specific serum IgG levels were measured by ELISA on DENV-coated plates (figure 1C). Both i.p. and i.f. immunization induced DENV-specific IgG, but the i.p. route induced a higher IgG response than the i.f. route. The virus-neutralizing capacity of the serum was assessed by plaque reduction neutralization test (PRNT_50_, figure 1D) and only i.p. immunization induced detectable levels of neutralizing Abs. Therefore, the i.p. immunization route was chosen for subsequent experiments.

### DENV2 E85-VRP immunization reduces viral load even during antibody-induced severe dengue disease

**Figure 2:**
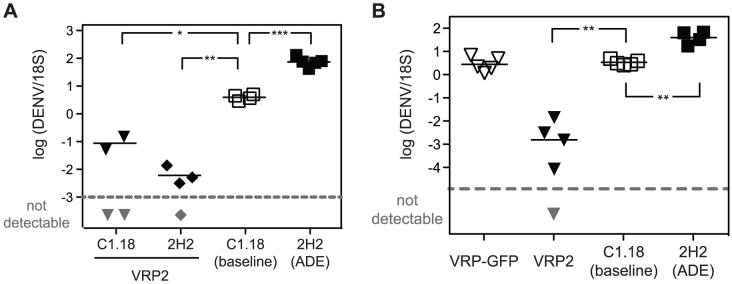
DENV2 E85-VRP-immunization reduced viral load during antibody-mediated severe dengue disease. AG129 mice were immunized with 1×10^6^ IU DENV2 E85-VRP i.p. 14 and 5 days prior to challenge with 5×10^8^ GE DENV. The baseline and ADE groups were included as described in figure 1. Viral RNA levels were measured in the liver 3 days after challenge. (A) One group of immunized mice was challenged in the presence of exogenous anti-DENV antibody 2H2 (black diamonds), and another group of immunized mice was challenged in the presence of a non-specific isotype control C1.18 (black triangles). (B) One group of mice was immunized with 1×10^6^ IU VRP expressing GFP (VRP-GFP, white triangles) instead of the DENV-E ectodomain (DENV2 E85-VRP, black triangles). Each symbol depicts one mouse, P-values from two-tailed unpaired t-test with Welch's correction, confidence interval 95%, * P≤0.05, ** P≤0.01, *** P≤0.001. Dotted line and symbols in gray as described in figure 1.

Next, to investigate whether immunization with DENV2 E85-VRP could also protect from Ab-induced severe dengue disease, AG129 mice were immunized with DENV2 E85-VRP as above. Subsequently, immunized mice were challenged with 5×10^8^ GE DENV in the presence (or absence) of exogenous anti-DENV Ab 2H2. Viral RNA levels were quantified in the liver on day 3 after challenge (figure 2A). DENV2 E85-VRP immunization reduced viral RNA levels to the same extent regardless of the presence or absence of exogenous anti-DENV Ab. As seen in figure 1 and in our previous studies [9], the viral RNA levels in the liver were about 10-fold higher in the ADE group as compared to the baseline group. To exclude a contribution from non-specific immune responses elicited by the VRP vector, AG129 mice were immunized with VRP expressing GFP (VRP-GFP) instead of the DENV-E protein. DENV2 E85-VRP-immunization, but not immunization with the non-specific VRP-GFP, reduced viral load in the liver on day 3 after challenge (figure 2B), demonstrating the specificity of the protection induced by DENV2 E85-VRP immunization. Taken together, these data demonstrate that immunization with DENV2 E85-VRP specifically reduces viral load upon DENV challenge, even in the presence of exogenous Ab (ADE).

### Passive transfer of serum from DENV2 E85-VRP-immunized mice prior to challenge can increase viral load upon infection

**Figure 3:**
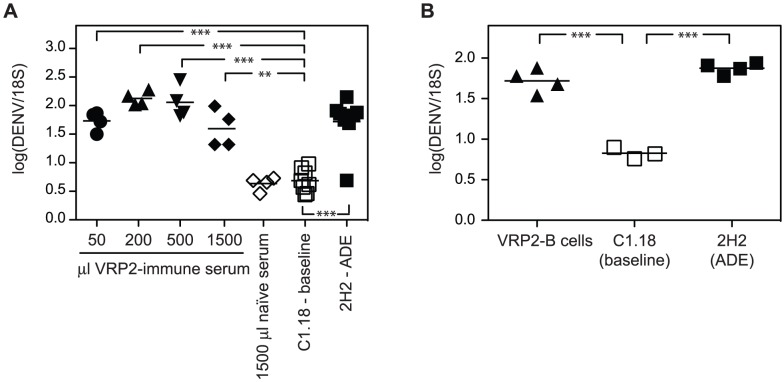
Passive transfer of DENV2 E85-VRP-immune serum or adoptive transfer of DENV2 E85-VRP-immune B cells can increase the viral RNA levels in the liver upon infection with DENV. (A) 50, 200 or 500 µl DENV2 E85-VRP-immune serum (from AG129 mice immunized i.p. 14 and 5 days prior to serum collection) were transferred i.v. into naïve AG129 recipient mice one day prior to challenge with 5×10^8^ GE DENV. One group received 500 µl of VRP immune serum i.v. 3, 2 and 1 day before challenge (total 1.5 ml). One control group received 1.5 ml naïve serum (500 µl 3, 2 and 1 day prior to challenge), another control group received no serum and was infected in the presence of anti-DENV Ab (ADE) and the last control group received no serum and was infected in the presence of an isotype control Ab of irrelevant specificity (baseline). Viral RNA was quantified in the liver by qRT-PCR 3 days after challenge. (B) 2×10^7^ DENV2 E85-VRP-primed splenic B cells (from AG129 mice immunized i.p. 14 and 5 days prior to B-cell isolation) were transferred i.v. into naïve AG129 recipient mice one day prior to challenge with 5×10^8^ GE DENV. The “baseline” and “ADE” control groups were included as in A. Each symbol depicts one mouse, P-values from two-tailed unpaired t-test with Welch's correction, confidence interval 95%, * P≤0.05, ** P≤0.01, *** P≤0.001.

To assess whether the protective effect of the DENV2 E85-VRP immunization was mediated by serum, 50 µl, 200 µl or 500 µl of serum from AG129 mice immunized 14 and 5 days earlier with 1×10^6^ IU of DENV2 E85-VRP were injected i.v. into naïve AG129 recipient mice one day prior to challenge with 5×10^8^ GE DENV. An additional group of recipients received a total of 1500 µl of DENV2 E85-VRP-immune serum i.v. (500 µl on day -3, -2 and -1). As controls, one group received 1500 µl naïve serum (500 µl on day -3, -2, -1) and two groups received no serum and were challenged either in the absence or presence of exogenous anti-DENV Ab (baseline and ADE groups). Viral RNA was quantified in the liver 3 days after challenge (figure 3A). Viral RNA levels in the liver were significantly higher in all groups that had received DENV2 E85-VRP-immune serum compared to the baseline group. As transfer of naïve serum had no effect on the viral load, we concluded that Ab present in the serum of immunized mice had caused ADE. To confirm these results, 2×10^7^ B cells from AG129 mice immunized with DENV2 E85-VRP 14 and 5 days earlier were adoptively transferred into naïve AG129 recipients one day prior to challenge with DENV. Similar to the ADE control animals, the mice that received DENV2 E85-VRP-primed B cells prior to challenge had viral RNA levels in the liver 3 days after challenge that were significantly higher than baseline (figure 3B). Taken together, these experiments have revealed that although immunization with DENV2 E85-VRP was protective, the presence of either serum or B cells from DENV2 E85-VRP-immunized mice did not reduce viral load upon challenge with DENV, but instead increased viral loads in the liver.

### CD8 T cells are required for protection after immunization with DENV2 E85-VRP 14 and 5 days prior to challenge

**Figure 4:**
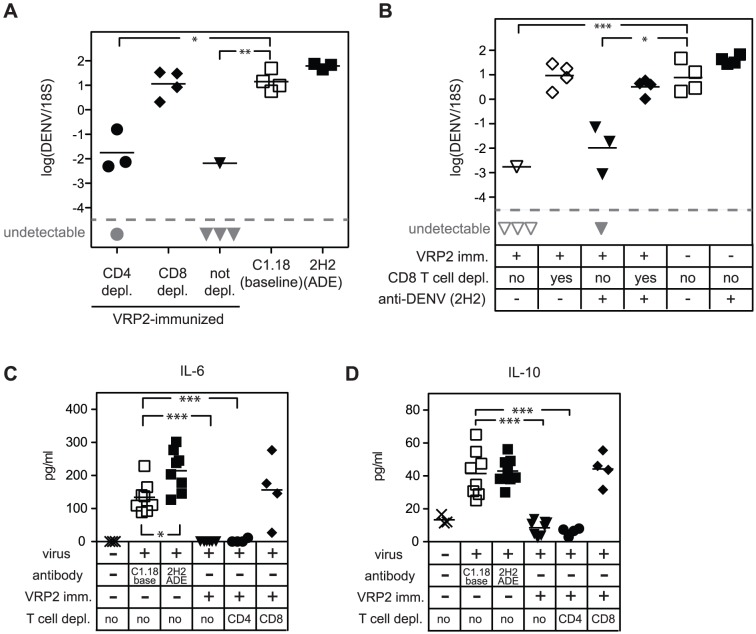
Contribution of T cells to protection from DENV challenge after DENV2 E85-VRP-immunization. (A and B) AG129 mice were immunized with 1×10^6^ DENV2 E85-VRP i.p. 14 and 5 days prior to challenge with 5×10^8^ GE DENV. The “baseline” and “ADE” groups were included. Viral RNA was quantified in the liver 3 days post infection. (A) Prior to challenge, CD4^+^ T cells (black circles) or CD8^+^ T cells (black diamonds) were depleted. This experiment was repeated twice with a total of 7–8 mice per group with similar results, one experiment is shown. (B) Mice were challenged with 5×10^8^ GE DENV either in the absence (open symbols) or in the presence (black symbols) of exogenous anti-DENV antibody. Half of the mice were depleted of their CD8^+^ T cell population prior to challenge (diamonds). (C and D) IL-6 and IL-10 were measured in the serum 3 days after challenge with virus in mice either not immunized or immunized as described in A. In addition, CD4^+^ or CD8^+^ T cells were depleted as indicated. Each symbol depicts one mouse, P-values from two-tailed unpaired t-test with Welch's correction, confidence interval 95%, * P≤0.05, ** P≤0.01, *** P≤0.001. Dotted line and symbols in gray as described in figure 1.

Thus far, our results have shown that immunization with DENV2 E85-VRP can protect from DENV challenge, and even prevent Ab-induced lethal dengue disease. With the chosen immunization schedule (day -14 and -5), immune serum or DENV2 E85-VRP-activated B cells had the potential, if transferred into naïve recipients, to increase viral load in the liver upon challenge. Therefore, we hypothesized that, using this particular schedule and route of immunization, T cells could be responsible for the DENV2 E85-VRP-mediated protection. To assess the contribution of T cells to protection, AG129 mice were immunized with DENV2 E85-VRP as described above, but prior to challenge with DENV, either CD4^+^ or CD8^+^ T cells were depleted. A control group was immunized but not depleted, and the baseline and ADE groups were included. As expected from previous experiments, DENV2 E85-VRP-immunization reduced viral RNA levels in the liver 3 days after challenge (figure 4A). CD4^+^ T cell-depletion had no detrimental effect on control of the liver viral RNA levels, but depletion of CD8^+^ T cells abrogated the reduction in liver viral RNA levels of the immunized mice. CD8^+^ T cell depletion also abolished the decrease in liver viral RNA levels observed in the immunized mice after DENV challenge in the presence of exogenous anti-DENV monoclonal Ab 2H2 (figure 4B).

The presence of elevated levels of multiple cytokines is a hallmark of severe dengue disease. To determine if cytokine levels were reduced in the immunized mice after challenge with DENV, serum levels of IL-6 and IL-10 were measured 3 days after infection. In addition, CD4^+^ or CD8^+^ T cells were depleted prior to challenge in some of the immunized mice (figure 4C and 4D). Serum IL-6 and IL-10 levels were lower in DENV2 E85-VRP-immunized mice than in non-immunized animals, probably due to the viral load reduction observed in immunized mice. Reduced cytokine levels were also observed in the serum of CD4^+^ T cell-depleted DENV2 E85-VRP-immunized mice. In contrast, levels of IL-6 and IL-10 in the serum of CD8^+^ T cell-depleted DENV2 E85-VRP-immunized mice were similar to non-immunized mice. Similar results were found for TNF, IFN-γ, IL-1β and KC/GRO (supplementary figure S1). Collectively, these results show that CD8^+^ T cells are responsible for the DENV2 E85-VRP-induced protection under this immunization protocol, as measured by reduction in liver viral RNA and serum cytokine levels on day 3 after challenge.

### DENV2 E85-VRP-primed wildtype T cells reduce viral RNA levels in the liver upon DENV challenge

**Figure 5:**
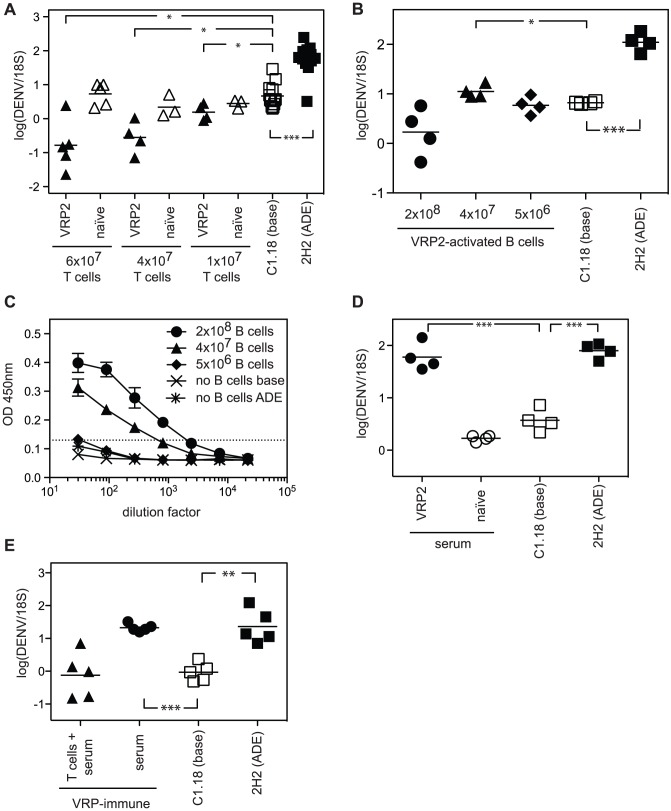
DENV2 E85-VRP-immune T cells from WT mice can reduce viral RNA levels in mice whereas DENV2 E85-VRP-immune serum from WT mice can enhance infection when transferred into AG129 mice. (A) WT129 mice were immunized on d-14 and d-5 with 1×10^6^ IU DENV2 E85-VRP i.p. Splenic T cells were isolated on day 0 and adoptively transferred into naïve AG129 recipients one day prior to challenge with 5×10^8^ GE DENV. Control groups include the “baseline” and “ADE” groups, as well as groups receiving naïve T cells. Viral RNA was quantified in the liver 3 days after challenge. (B, C) 2×10^8^, 4×10^7^ or 5×10^6^ B cells from WT mice immunized with DENV2 E85-VRP (d-14 and -5) as described above were transferred into naïve AG129 mice one day prior to challenge with DENV (as above). The “baseline” and “ADE” groups received no B cells. Viral RNA was quantified in the liver 3 days after challenge (B) and serum levels of DENV-IgG was measured by ELISA 3 days after challenge (C). (D) Serum (200 µl) from WT mice immunized with DENV2 E85-VRP (d-14 and -5) as described above was passively transferred into naïve AG129 mice one day prior to challenge with DENV (as above). Viral RNA was quantified in the liver 3 days after challenge. (E) Serum (200 µl) from WT mice immunized with DENV2 E85-VRP (d-14 and -5) was transferred into naïve AG129 mice with or without 4×10^7^ T cells from WT mice immunized with DENV2 E85-VRP (d-14 and -5) one day prior to challenge with DENV2. Viral RNA was quantified in the liver 3 days after challenge. Each symbol depicts one mouse, except for C where n = 4 for the groups receiving B cells and 2 for the groups receiving no B cells (base and ADE), P-values from two-tailed unpaired t-test with Welch's correction, confidence interval 95%, * P≤0.05, ** P≤0.01, *** P≤0.001. The dotted line represents the limit of detection of the assay.

To confirm the results obtained with AG129 mice, in which DENV2 E85-VRP-induced immune response was elicited in the absence of type I and II IFN receptors, congenic WT mice were immunized on day -14 and -5 with DENV2 E85-VRP i.p. Splenic T or B cells were isolated from DENV2 E85-VRP-immunized WT mice on day 0 (MACS negative selection of total T or B cells) and adoptively transferred into naïve AG129 one day prior to challenge with DENV. The viral RNA was quantified in the liver 3 days after challenge. This experimental setup allowed us to assess how DENV2 E85-VRP-primed WT T or B cells contributed to protection upon DENV infection. AG129 mice were used as DENV-sensitive recipients, thereby allowing us to assess the protective versus pathogenic capacity of the transferred WT T or B cells under stringent challenge conditions. Three different amounts of DENV2 E85-VRP-primed T cells (or naïve T cells) from WT mice were transferred into AG129 recipients. All the recipient groups that received DENV2 E85-VRP-primed T cells prior to challenge with DENV had lower levels of viral RNA in the liver 3 days after challenge (figure 5A, black triangles), whereas animals that received naïve WT T cells had viral RNA levels similar to the baseline group (figure 5A, white triangles). These results demonstrate a protective role for the VRP vaccine-induced WT T cells during DENV infection.

### Transfer of DENV2 E85-VRP-primed WT B cells can reduce or increase the liver viral RNA levels depending on the number of B cells transferred; and DENV2 E85-VRP-immune WT serum can increase the liver viral RNA level

Three different numbers of DENV2 E85-VRP-primed B cells from WT mice (2×10^8^, 4×10^7^ or 5×10^6^ B cells) were transferred into naïve AG129 recipients one day prior to challenge. Transfer of 2×10^8^ DENV2 E85-VRP-primed WT B cells reduced the liver day 3 viral RNA levels in 3 out of 4 animals compared to the animals that received no B cells (baseline), but this difference was not statistically significant when the whole group was considered (figure 5B). Transfer of 4×10^7^ DENV2 E85-VRP-primed WT B cells caused a small but significant increase in the viral load in the liver on day 3, likely via ADE. Transfer of 5×10^6^ B cells had no effect on the viral load, probably due to the low amount of B cells transferred. To verify that the transferred B cells produced virus-specific Abs, DENV2-specific IgG levels were measured by ELISA on DENV-virion-coated plates in serum obtained from recipients 3 days after challenge (Figure 5C). No DENV2-specific IgG was detected in the mice that had not received B cells and were challenged with DENV on day 0 (either with or without exogenous anti-DENV Ab). In the groups that received WT DENV2 E85-VRP-primed B cells one day prior to challenge, the amount of Ab detected 3 days after challenge was proportional to the number of B cells transferred. DENV-specific IgG could not be detected by ELISA in the group that received only 5×10^6^ WT DENV2 E85-VRP-primed B cells, probably due to the low amount of B cells transferred.

To confirm the results obtained from transfer of DENV2 E85-VRP-primed WT B cells, we performed a passive transfer experiment using DENV2 E85-VRP-immune serum from WT mice. WT mice were immunized on d-14 and -5 with 1×10^6^ IU of DENV2 E85-VRP i.p. followed by collection of serum on day 0 and transfer of the immune serum into naïve AG129 mice one day prior to challenge with DENV. Viral RNA levels were quantified in the liver 3 days after challenge (figure 5D). Mice that received 200 µl of DENV2 E85-VRP-immune WT serum had elevated DENV RNA levels in the liver compared to mice that received either naïve serum, or no serum (baseline).

Next, we assessed the effect of adoptively transferring DENV2 E85-VRP-primed WT T cells together with an enhancing amount of DENV2 E85-VRP-immune WT serum into AG129 recipients. WT mice were immunized with 1×10^6^ IU DENV2 E85-VRP i.p. on days -14 and -5, and on day 0 serum was collected and total splenic T cells were isolated by negative selection. 200 µl of DENV2 E85-VRP-immune WT serum was transferred with or without 4×10^7^ DENV2 E85-VRP-primed WT T cells into naïve AG129 recipient mice one day prior to challenge with DENV2. As shown in figure 5E, transfer of serum alone increased viral RNA levels in the liver on day 3 after challenge, but co-transfer of T cells and serum did not increase liver viral RNA levels.

Taken together, these data confirm the results obtained from our studies using AG129 mice. Studies with both AG129 and WT mice have demonstrated that DENV2 E85-VRP-immune T cells can reduce viral load, whereas DENV2 E85-VRP-immune serum induced by the day -14 and -5 immunization schedule, if taken in isolation from other components of the immune system, can increase viral load upon challenge.

### DENV2 E85-VRP immunization confers long-term protection against DENV challenge

**Figure 6:**
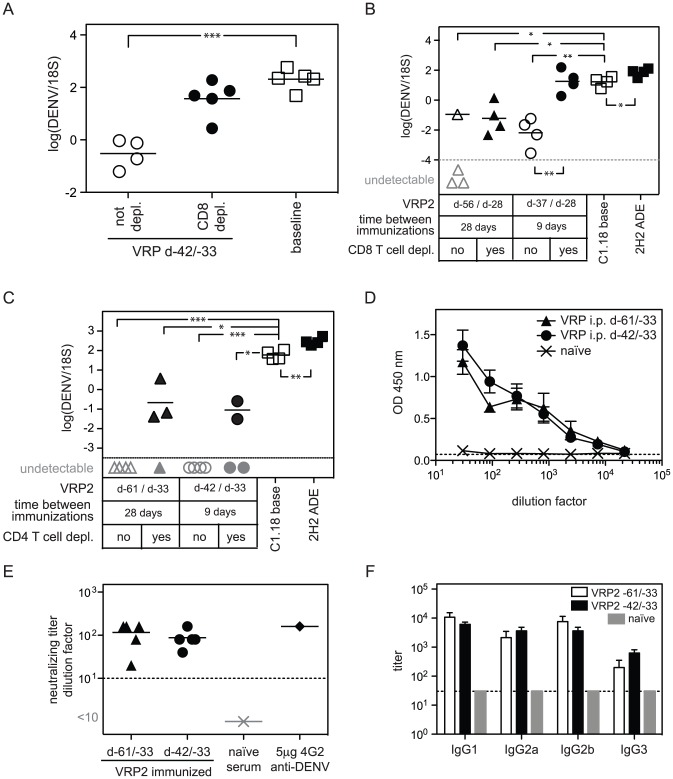
DENV2 E85-VRP provides long-term protection; and the interval between the two immunizations modulates the degree to which the vaccine-induced protection relies on CD8^+^ T cells. (A) AG129 mice were immunized with 1×10^6^ IU DENV2 E85-VRP i.p. 42 and 33 days before challenge with 5×10^8^ GE DENV. Half of the mice were depleted of their CD8^+^ T cell population (black circles), and one group was left untreated before challenge (baseline). Viral RNA was quantified in the liver 3 days after challenge. (B) AG129 mice were immunized twice with 1×10^6^ IU DENV2 E85-VRP i.p. either 28 or 9 days apart. 28 days after the second immunization, mice were challenged with 5×10^8^ GE DENV. Half of the mice were depleted of their CD8^+^ T cell population. Viral RNA was quantified in the liver 4 days after challenge. (C) AG129 mice were immunized as in B (28 or 9 days apart). 33 days after the second immunization, mice were challenged with 5×10^8^ GE DENV. Before challenge, half of the mice were depleted of their CD4^+^ T cell population. Viral RNA was quantified in the liver 3 days after challenge. (D, E, F) AG129 mice were immunized with 1×10^6^ IU DENV2 E85-VRP i.p. 28 days apart (-61/-33) or 9 days apart (-42/-33), and on day 0, serum levels of DENV-specific IgG were measured by ELISA (D), neutralizing titers were determined by PRNT_50_ (E), and DENV-specific IgG1, IgG2a, IgG2b and IgG3 were measured by ELISA (F). Each symbol depicts one mouse, except for D where n = 4 for the groups receiving B cells and 2 for the groups receiving no B cells (base and ADE) and F where n = 5 for experimental groups, naïve serum was included as a negative control, P-values from two-tailed unpaired t-test with Welch's correction, confidence interval 95%, * P≤0.05, ** P≤0.01, *** P≤0.001. Dotted line and symbols in gray as described in figure 1.

We have shown that DENV2 E85-VRP-immunization confers CD8^+^ T cell-mediated short-term protection against DENV challenge. To examine whether DENV2 E85-VRP-immunization can confer longer-term protection against DENV challenge, AG129 mice were immunized twice with 1×10^6^ IU of DENV2 E85-VRP i.p. 9 days apart (as described in all experiments so far), and mice were challenged with DENV 33 days after the second immunization. Half of the immunized mice were depleted of CD8^+^ T cells before challenge. Three days after challenge, viral RNA levels in the liver were significantly lower in the immunized mice compared to the non-immunized baseline group, and CD8-depletion abrogated this decrease in viral load (figure 6A). Thus, DENV2 E85-VRP immunization can provide CD8^+^ T cell-dependent, long-term protection against DENV, as determined by reduction of liver viral RNA titer 3 days post challenge in AG129 mice.

### The time-interval between the two immunizations modulates the relative contribution of cellular and humoral immunity to protection

Two immunizations with 1×10^6^ IU DENV2 E85-VRP i.p. 9 days apart provided CD8^+^ T cell-dependent long-term protection against DENV challenge. However, the 9-day interval between the two immunizations (as in all experiments so far) may not be long enough to induce an optimal Ab response. Therefore, we increased the time interval between the immunizations to investigate whether this would modify the relative contribution of cellular and humoral immunity to long-term protection.

AG129 mice were given two immunizations with 1×10^6^ IU of DENV2 E85-VRP i.p. 28 or 9 days apart. Mice were challenged with DENV 28 days after the second immunization and in each group, some mice were depleted of their CD8^+^ or CD4^+^ T cells prior to challenge. Viral RNA levels were measured in the liver 4 days after challenge (figure 6B, C). Livers were harvested 4 days after challenge instead of 3 (as in the short-term experiments performed thus far) because in the long-term experiments, mice are older and heavier, and the viral load increase caused by exogenous anti-DENV Ab (ADE) starts to be detectable 4 days post infection (our unpublished observations). Baseline and ADE groups were included as controls. All immunized and CD8^+^ T cell-competent mice had lower viral RNA levels in the liver on day 4 compared to non-immunized mice (baseline) (figure 6B). CD8^+^ T cell-depleted mice that were immunized 28 days apart also contained lower viral RNA levels than the baseline group, indicating minimal requirement for CD8^+^ T cells in protection mediated by the 28-day apart immunization protocol, possibly due to further maturation of the Ab response during the extended interval. In contrast, CD8^+^ T cell-depleted mice that were immunized 9 days apart had similar levels of viral RNA as the baseline mice, indicating a critical role for CD8^+^ T cells in protection induced by the 9-day apart immunization protocol. This result suggests that reducing the time between immunizations may increase the dependency on CD8^+^ T cells for protection, possibly due to sub-optimal Ab maturation when the time between immunizations is insufficient. Regardless of the immunization protocol (28 or 9 days apart), depletion of CD4^+^ T cells prior to challenge with DENV had only a minimal (and not significant) effect on protection (figure 6C).

To determine if the different immunization schedules had an influence on the induction of DENV-specific Ab, the serum of mice immunized 28 days (d-61/-33) or 9 days (d-42/-33) apart was analyzed 33 days after the second immunization (but before challenge with DENV). No difference in DENV-specific IgG levels was observed by ELISA (figure 6D), and neutralizing titers were similar as determined by PRNT_50_ (figure 6E). Titers of DENV-specific IgG1, IgG2a, IgG2b and IgG3 were also similar between the two immunization protocols (figure 6F), suggesting that other characteristics of the Ab response (e.g. epitope-specificity, affinity/avidity, and biological activities such as ADCC and complement fixation) induced by the extended immunization schedule likely account for protection.

## Discussion

In this study, alphavirus VRP expressing the DENV2-E protein ectodomain (DENV2 E85-VRP) were used to immunize mice prior to challenge with DENV in order to assess the relative contribution of humoral and cellular immunity in a protective vaccine-induced immune response to DENV. A better understanding of the determinants of protection after dengue vaccination is urgently needed [14], especially after the recent report of the first phase IIb clinical trial of a tetravalent live-attenuated dengue-vaccine candidate [12]. For reasons that remain unclear, the phase IIb trial showed only limited efficacy of the vaccine despite induction of a balanced Ab response against all four serotypes. Our study begins to address this lack of knowledge by assessing the relative role of the humoral and cellular arms of a protective vaccine-induced immune response.

Two immunizations with DENV2 E85-VRP (9 days apart, day -14 and -5) dramatically reduced the viral RNA levels in the liver 3 days after challenge with DENV; viral RNA was undetectable in some immunized animals. All immunized mice survived at least 20 days post challenge, and 80% survived without showing any sign of disease until the experiment was terminated on day 60. Immunization with DENV2 E85-VRP reduced the viral load upon challenge even in the presence of sub-protective, enhancing levels of exogenous anti-DENV Ab. A regimen of two immunizations with DENV2 E85-VRP, either 9 or 28 days apart, was protective up to 33 days after the second immunization, the latest time point tested in this study. These findings are remarkable considering that AG129 are highly susceptible to DENV replication, and that a DENV dose as low as 5×10^4^ GE (approximately 1 PFU) causes paralysis in 100% of the mice by day 25 post-infection [22]. Thus, immunization with DENV2 E85-VRP confers robust protection and appears to mediate near-sterilizing immunity.

T cell depletion and adoptive transfer experiments demonstrated that when mice were challenged 5 days after the second immunization (immunization on day -14/-5), CD8^+^ T cells were responsible for reducing the viral load upon challenge. When mice were challenged more than 4 weeks after the second immunization, protection appeared to be dependent on CD8^+^ T cells in mice immunized 9 days apart, but independent of CD8^+^ T cells in mice immunized 28 days apart. In all experiments performed, DENV2 E85-VRP-primed T cells were protective as determined by decreased viral RNA levels in the liver and reduced cytokine levels 3 days after challenge. In contrast, transfer of DENV2 E85-VRP-immune serum or DENV2 E85-VRP-immune B cells had the potential to increase liver viral RNA levels. When T cells and serum were co-transferred, no enhancement was observed.

The use of AG129 mice (lacking type I and II IFN receptors) in this study is not ideal as the mechanisms by which DENV causes disease in these mice may differ relative to WT animals. However, the experiments described in this study would be impossible in WT mice, as, to this day, there are no known DENV strains that replicate and cause disease in WT mice. DENV inhibits IFN signaling in humans but not in mice [24]–[26], possibly explaining why WT mice are resistant to DENV infection and disease. The AG129 mouse model of DENV infection is the most thoroughly characterized animal model of DHF/DSS-like disease to date. This mouse model recapitulates many key features of the human disease including vascular leakage, cytokine release, high viremia, low platelet counts and elevated hematocrit [8], [9]. AG129 mice have been used to demonstrate ADE [8], [9] and to evaluate the therapeutic efficacy of modified antibodies that no longer bind to the Fcγ-receptor [27].

The results obtained by immunization and challenge of AG129 mice were confirmed with transfer experiments in which DENV2 E85-VRP-immunized WT mouse T cells, B cells, or serum were transferred into naïve AG129 recipients prior to challenge with DENV. This experimental approach was chosen to assess under stringent challenge conditions the efficacy of a DENV2 E85-VRP-induced immune response generated in WT (i.e. IFN receptor competent) animals. Additionally, the transfer approach allowed us to separate the cellular and the humoral components of a protective vaccine-induced immune response and evaluate their relative contribution to protection versus disease enhancement. The presence of DENV2 E85-VRP-primed WT T cells reduced viral load upon challenge with DENV; whereas WT serum or DENV2 E85-VRP-primed WT B cells had the potential to increase viral RNA levels at certain concentrations. When present in a sufficiently high concentration, DENV2 E85-VRP primed WT B cells reduced viral load.

One of the challenges to developing a safe and efficient vaccine against DENV is that a vaccine should simultaneously generate a protective immune response against all 4 DENV serotypes. An unbalanced immune response resulting in sub-protective levels of Ab against one of the serotypes could potentially result in Ab-mediated disease enhancement [2]. In theory, a vaccine-induced Ab response that is initially protective could wane over time and reach levels at which Ab exacerbates disease, as even neutralizing Abs can cause ADE at lower, sub-protective concentrations [9]–[11]. Although passive transfer of serum from DENV2 E85-VRP-immune mice did not reduce viral load upon challenge in our study, in another study, BALB/c females immunized with VRP co-expressing DENV-E- and prM-protein could passively transfer antibodies to their pups, which were subsequently protected from a lethal intracranial challenge with DENV2 strain NGC [20]. It is also important to note that in our long-term experiments, two immunizations with DENV2 E85-VRP 28 days apart reduced DENV viral RNA titer in the liver upon challenge in the absence of CD8^+^ or CD4^+^ T cells. This strongly suggests that vaccination with DENV2 E85-VRP can readily induce a protective antibody response in AG129 mice when another immunization schedule is chosen. In our study, T cell responses were beneficial for the host and co-transfer of immune T cells together with immune serum abrogated the immune serum-mediated enhancement. Therefore, we propose that a vaccine triggering both the humoral and the cellular arms of the immune system may be more efficient and safer than a vaccine relying exclusively on the induction of Ab.

DENV2 E85-VRP induced a highly protective T cell response although the DENV E-protein is not a major T cell target during natural DENV infection [28]–[30]. A likely explanation for the protective T cell responses elicited by the VRP vaccination is the excellent ability of VRPs to induce CD8^+^ T cell responses [31]. VRPs efficiently target and activate dendritic cells [32] and are known to induce both humoral and cellular immunity to the encoded transgene [31]. In addition, VRPs not encoding a transgene have potent adjuvant ability when co-injected with antigen [33] and strongly amplify the CD8^+^ T cell responses to the co-delivered antigen [34]. During a natural infection with DENV, the T cell response to non-structural antigens is dominant [30], [35] and may outcompete the response to structural proteins. As the DENV2 E85-VRP does not code for DENV non-structural proteins, the T cell response to the E-protein may be higher than what would be expected after natural infection.

The relative contribution of CD8^+^ T cells to the VRP vaccine-mediated protection in our murine model was influenced by the interval between immunizations. In the experiments where mice were challenged four weeks after the second immunization, two doses of DENV2 E85-VRP given 9 days apart protected via the induction of CD8^+^ T cells, whereas if the doses were given 28 days apart, CD8^+^ T cells were unnecessary for the vaccine-induced protection. The interval of 9 days between the two immunizations may not induce an Ab response that is sufficient to protect from challenge in the absence of CD8^+^ T cells. However, the 28-day interval immunization schedule provides protection even in the absence of CD8^+^ T cells, possibly through a more complete maturation of humoral immunity. This suggests that a robust CD8^+^ T cell response may be crucial for protection if the Ab response is insufficient and/or inefficient. Both protocols induced similar levels of anti-DENV Ab responses, as measured by ELISA and PRNT_50_, but only the 28-day protocol protected from challenge in the absence of CD8^+^ T cells. Therefore, the presence of neutralizing Abs in the serum before challenge (as measured by PRNT_50_) did not correlate with protection in vivo. Similarly, immunization with DENV2 E85-VRP via the i.p. or the i.f. routes induced short-term protection when mice were challenged, but only the i.p. route induced neutralizing Ab responses. These results support the emerging notion that measuring neutralizing Abs by PRNT_50_ may not accurately predict the efficacy of a vaccine against DENV [36]–[38], and highlights the urgent need for further investigation into the correlates of protection against DENV.

Based on our results, the lack of induction of a robust anti-DENV T cell response may be a potential explanation for the recent results of a phase IIb clinical trial of a live attenuated tetravalent dengue vaccine showing low efficacy against DENV2 despite the induction of DENV2-specific neutralizing Abs [12]. The vaccine used in the clinical trial consisted of a yellow fever backbone, and therefore did not contain DENV non-structural proteins, the major targets of CD8^+^ T cells during natural infection in humans. The cellular responses were possibly skewed towards the yellow fever backbone non-structural proteins. Unlike the VRPs used in our study, which induced a protective cellular response to the E-protein, the live attenuated vaccine used in the phase IIb trial may not have triggered a strong T cell response to the DENV E proteins. The protective T cell responses to DENV E-protein observed in our study may be the direct result of the remarkable ability of the VRPs to trigger CD8^+^ T cell responses to the encoded transgene.

Cellular immunity during DENV infection is often perceived as minimally protective [2], or potentially pathogenic [13], although a protective role for T cells has been demonstrated previously in mouse models of DENV infection [35], [39]. The present study investigated the contribution of different amounts of homologous T cells to protection. Our results showed that the presence of homologous T cells was always beneficial to the host, and did not increase the severity of disease or the viral load. Further studies are now needed to determine whether heterologous T cells behave similarly to homologous T cells, or whether they contribute to pathogenesis. In addition, the mechanisms by which CD8+ T cells mediate protection after DENV2 E85-VRP vaccination need to be clarified.

Our model is well suited to investigate the relative contribution of humoral and cellular immunity to protection or pathogenesis after vaccination or during sequential infections with DENV. Our approach makes it possible to isolate serum, B cells and/or T cells and assess their respective roles in vivo, either alone or in combination. However, extrapolation to dengue vaccination in humans should be done with great caution, as is the case with any finding made using an animal model. Our findings thus delineate areas that deserve thorough exploration in future human studies. They also highlight the need to take a comprehensive approach that considers the roles of both humoral and cellular immunity in order to tackle the challenges posed by the development of a dengue vaccine [36].

In summary, despite several dengue vaccine candidates in phase I, II and III clinical trials, little is known about the immunological mechanisms of protection (or potential enhancement) after dengue vaccination. This study starts to explore the mechanisms of dengue vaccine-mediated protection or enhancement by examining the relative contribution of the humoral and cellular arms of the immune system during a protective vaccine-induced immune response to DENV. Our results demonstrate that the humoral component of a protective vaccine-induced immune response to DENV had the potential, when taken in isolation from other components of the immune system, to reduce or increase viral load upon challenge, whereas cellular immunity, alone or in combination with humoral immunity, was always beneficial to the host. These findings suggest that the role of T cells in the context of DENV vaccination should not be ignored, and that a safe and efficient vaccine against DENV should ideally trigger both arms of the immune system.

## Materials and Methods

### Ethics statement

This study was carried out in strict accordance with the recommendations in the Guide for the Care and Use of Laboratory Animals of the National Institutes of Health, the US Public Health Service Policy on Humane Care and Use of Laboratory Animals, and the Association for Assessment and Accreditation of Laboratory Animal Care International (AAALAC). All experimental procedures were approved and performed according to the guidelines set by the La Jolla Institute for Allergy and Immunology Animal Care and Use Committee (protocol number AP-28SS1-0809).

### Mouse experiments

129/Sv mice deficient in type I and II interferon receptors (AG129, originally obtained from Dr. Skip Virgin at Washington University in St. Louis) and wild-type 129/Sv mice (purchased from Taconic) were housed under SPF conditions at the La Jolla Institute for Allergy and Immunology (LIAI). For all experiments, sex-matched 5 to 6 week-old mice were used. For challenge experiments, mice were infected intravenously (via the tail vein) with 5×10^8^ GE of DENV serotype 2, strain S221 diluted in a total volume of 200 µl PBS with 10% FCS. For survival studies, mice were sacrificed when moribund or at the first signs of paralysis.

### Viral stocks production

DENV strain S221 (serotype 2) is a triple plaque-purified clone from the D2S10 quasi-species population [23]. S221 was amplified in C6/36 cells (purchased from ATCC) cultured at 28°C in Leibovitz's L-15 medium (Gibco) supplemented with penicillin, streptomycin, HEPES, and 10% FBS as previously described [40]. Genomic equivalents (GE) were quantified by real-time qRT-PCR as previously described [40]. Based on a standard baby hamster kidney cell (BHK-21) plaque assay, there are approximately 5×10^4^ GE/PFU for S221.

### Antibodies

2H2 is an IgG2a reactive for the prM/M protein of DENV, serotypes 1–4 (IgG2a anti-DENV1-4 prM). 2H2 hybridoma was purchased from ATCC and grown in PFHM-II (Gibco) with penicillin (100 U/ml), streptomycin (100 µg/ml) and 55 µM β-mercaptoethanol in CELLine CL1000 bioreactors (Wilson Wolf Manufacturing Corporation). Antibody was purified using protein G-coupled resin according to the manufacturer's instructions (Pierce), dialyzed against PBS, concentrated, and sterile filtered prior to use in experiments. The purity of Ab preparations was verified by SDS-PAGE and binding to DENV was assessed by ELISA. Protein content was quantified using a BCA protein assay kit (Pierce). Antibody C1.18, mouse IgG2a isotype control, was purchased from BioXCell.

### Viral RNA quantification

Tissues were collected into RNAlater (Qiagen) and subsequently homogenized as described previously [40]. RNA was isolated and DENV and relative 18S were quantified using real-time qRT-PCR as described previously [40].

### T cell depletions

T cell-depleting antibodies 2.43 (IgG2b anti-mouse CD8), GK1.5 (IgG2b anti-mouse CD4) and the isotype control LTF2 (IgG2b) were purchased from BioXCell. CD8^+^ or CD4^+^ T cells were depleted by administering 250 µg of 2.43 or GK1.5 antibody intraperitoneally in 200 µl total volume in PBS 3 days and 1 day before challenge with virus.

### DENV ELISA

Sucrose-purified DENV2 strain S221 was used to coat 96-well plates overnight at 4°C (10^9^ GE per well in 50 µl 0.1 M NaHCO_3_). Next, virus on plates was UV-inactivated and plates were blocked with 100 µl Blocker Casein in PBS (Thermo Scientific, 1 hour, room temperature). Blocking solution was flicked off and serum was titrated 1∶3 over 7 titration steps, starting with an initial dilution of 1 in 30 in PBS. Serum dilutions were incubated 1.5 hr at room temperature, followed by washing of the plates three times with 0.05% (v/v) Tween 20 (Sigma) in PBS (Gibco). Bound antibody was detected using a 1∶5000 dilution of HRP-conjugated goat anti-mouse IgG, IgG1, IgG2a, IgG2b or IgG3 antibodies (Jackson Immunoresearch) and TMB (eBioscience). Results are reported as a plot of absorption (OD 450 nm) versus dilution. Alternatively, in the ELISA measuring the DENV-specific IgG1, IgG2a, IgG2b, IgG3, the titer was reported as the greatest dilution with an absorption higher than twice the background absorption.

### Neutralizing activity of serum (plaque reduction neutralization test 50%, PRNT_50_)

One day prior to infection, a total of 1×10^5^ BHK cells were seeded in each well of standard 24-well plates (Costar) in 1 ml of medium (1×10^5^ cells/ml cell suspension in MEM supplemented with 10% FCS, 10 mM HEPES Buffer, 100 U/ml Penicillin, 100 µg/ml Streptomycin, all from Gibco). On the day of the assay, 20 µl of serum was diluted in 80 µl culture medium and subsequently titrated 1∶2 over 6 titration steps. 50 µl of each serum dilution was mixed with 50 µl of DENV2 (7.5×10^7^ GE/ml in medium) and incubated 1 hr at 4°C. Each serum/virus mix (total volume of 100 µl) was used to infect BHK cells in one well of the 24-well plates seeded the day before. The medium was carefully removed from the monolayer of cells prior to infection, and 100 µl of the serum/virus mix was applied to the monolayer of cells in each well. For each serum sample, the dilutions tested were therefore 1∶10, 1∶20, 1∶40, 1∶80, 1∶160 and 1∶320. Plate were subsequently incubated for 1 hour at 37°C (with 5% CO_2_). After 1 hour, the infection medium was removed and each well was overlaid with 1 ml of culture medium (as described above) containing 1% (w/v) Carboxymethylcellulose (CMC) Medium Viscosity (Sigma). Following incubation of the plates for 3 days at 37°C (with 5% CO_2_), cells were fixed with 1 ml per well of a 4% buffered formalin solution (Fisher Scientific) for 30 minutes at room temperature. Overlay/formalin mix was decanted, the cells were washed 3 times with PBS, and the cell layer was stained with 1% crystal violet (Fisher Scientific) in 20% ethanol. Plaques were counted and the highest dilution neutralizing 50% of plaques or more was reported as neutralizing titer (PRNT_50_). As a positive control, 10 µg of the DENV-neutralizing monoclonal Ab 4G2 (IgG2a anti-DENV1-4 E) was used and titrated 1∶2 like the serum samples.

### Adoptive transfers

Donor spleens were homogenized through 70 µm strainers and the desired cell populations were isolated by MACS negative selection (depletion of non-target cells, keeping the desired population untouched) using the Pan B Cell Isolation Kit or Pan T Cell Isolation Kit II from Miltenyi Biotech. Procedures were performed according to the manufacturer's instructions. Cells were enriched to over 90% purity (T cells) and 80% purity (B cells) as determined by flow cytometry. Cells were administered intravenously into recipient mice one day prior to challenge with DENV.

### Cytokine measurements

Cytokine levels in the serum were measured using the mouse pro-inflammatory 7-plex base kit (IFN-γ, IL-1β, IL-6, IL-10, IL-12p70, KC/GRO/CINC, TNF) from MDS Meso Scale Discovery according to the manufacturer's instructions.

## Supporting Information

Figure S1 AG129 mice were immunized with 1×10^6^ DENV2 E85-DENV2 E85-VRP i.p. 14 and 5 days prior to challenge with 5×10^8^ GE DENV. Some groups were depleted of their CD4^+^ or CD8^+^ T cell populations as indicated. Baseline and ADE groups were included. Three days after challenge, levels of TNF (A), IFN-γ (B), IL-1β (C) and KC (D) were measured in the serum. Each symbol depicts one mouse, P-values from two-tailed unpaired t-test with Welch's correction, confidence interval 95%, * P≤0.05, ** P≤0.01, *** P≤0.001.
(EPS)
